# Healthcare staff experiences on the impact of COVID-19 on emergency departments: a qualitative study

**DOI:** 10.1186/s12913-024-11362-9

**Published:** 2024-08-12

**Authors:** Ahmet Butun

**Affiliations:** Faculty of Health Sciences, Mardin Artuklu University, Kampus yerleskesi, Artuklu/Mardin, Mardin, 47000 Turkey

**Keywords:** Emergency departments, Emergency care services, COVID-19, Pandemic, Healthcare staff, Qualitative

## Abstract

**Background:**

The COVID-19 pandemic has had a major impact on the access and delivery of healthcare services, posing unprecedented challenges to healthcare staff worldwide. Frontline healthcare staff faced unique stressors and challenges that impact their well-being and patient care. This qualitative study aimed to explore the experiences and perspectives of frontline ED healthcare staff on emergency care services during the COVID-19 pandemic, providing valuable insights into the challenges, adaptations, and lessons learned in delivering emergency care.

**Methods:**

This study utilized a qualitative approach. In-depth semi-structured interviews were conducted with 30 ED healthcare staff from three different hospitals located in Turkey between 15/03/2022 and 30/04/2022. Convenience sampling was used to recruit participants. The duration of the interviews ranged from 28 to 37 min. Data saturation was reached as no new information was gathered. The data were analyzed using the thematic analysis method. NVivo software was used to manage the data analysis process. Member check was carried out to ensure that the generated themes conformed to the participants’ views.

**Results:**

15 sub-themes under three themes emerged: (1) the impact of COVID-19 on emergency care services, including sub-themes of “introducing a COVID-19 unit in the ED”, “changes in the routine functioning of EDs”, “changes in the number of ED visits”, “quality of care”, “resources”, and “increased workload”; (2) the psychological effects of COVID-19 on ED healthcare staff, including sub-themes of “staying away from family”, “fear”, “society’s perspective on healthcare professionals”, “morale-staff burnout”, “psychological and emotional effects”, and “unable to receive sufficient support”; and (3) the difficulties faced by ED healthcare staff, including sub-themes of “difficult working conditions”, “community-based effects difficulties”, and “COVID-19 is an unknown situation”.

**Conclusion:**

Staff burnout threatens the quality of patient care and staff retention, and therefore this should be addressed by ED directors and leaders. This study could inform appropriate stakeholders regarding lessons learned from COVID-19 to better manage future pandemics. Learning from such lived experiences and developing appropriate interventions to minimize the difficulties faced during COVID-19 would allow better management of future pandemics. This study calls for a reform to address the challenges faced by healthcare staff, improve the overall response to public health crises, and enhance the resilience of healthcare systems for future crises.

## Background

The COVID-19 pandemic has had a major impact on the access and delivery of healthcare services, posing unprecedented challenges to healthcare staff worldwide. The COVID-19 pandemic caused a high mortality rate and disruption to healthcare and social care around the world [1, 2]. The pandemic has required the reconfiguration of health services to ensure the maintenance of essential health services [3]. The COVID-19 pandemic also caused delays in seeking care due to the fear of infection [4]. Delaying care may increase morbidity and mortality in non-COVID-19 patients [2, 5]. The first COVID-19 case was identified on 11 March 2020 in Turkey. Around 35 million people were tested, around 2.9 million patients were diagnosed with COVID-19, around 30.000 patients were died within 1 year of starting of the pandemic, between 11 March 2020 and 11 March 2021 [6].

Emergency Departments (EDs) healthcare staff face an enormous mental burden and physical exertion when caring for patients potentially infected with COVID-19. Frontline healthcare staff face unique stressors and challenges that impact their well-being and patient care [7, 8]. ED healthcare staff were deeply impacted by COVID-19, and they are at a high risk of burnout due to COVID-19 [9, 10]. However, the COVID-19 pandemic has underscored the need for qualitative research to understand the experiences and perceptions of healthcare staff, as well as the use of healthcare services during this unprecedented health emergency [11–13]. Therefore, exploring the experiences of ED healthcare staff throughout the COVID-19 pandemic will provide a comprehensive understanding of their unique experiences and challenges.

In addition, studies showed that EDs in low-income and middle-income countries are likely to be impacted more significantly by the effects of the COVID-19 pandemic than those in high-income countries [9, 14, 15]. Thus, studying how the COVID-19 pandemic affects emergency care services in the context of a middle-income country (Turkey) is needed. This study focused on the impact of the COVID-19 pandemic on the general working conditions of the emergency care system, including ED services, ED healthcare staff, and ED patients in addition to its impact on general healthcare system. This qualitative study aims to explore the experiences and perspectives of frontline ED healthcare staff on emergency care services during the COVID-19 pandemic, providing valuable insights into the challenges, adaptations, and lessons learned in delivering emergency care.

## Methods

### Study design

This study utilized a qualitative exploratory descriptive approach as this allows to explore, describe, and a deep understanding of ED healthcare staff experiences during the COVID-19 pandemic.

### Participants and settings

Participants were ED healthcare staff including ED nurses (*n* = 20), ED physicians (*n* = 8), and ED consultants (*n* = 2) from 3 different hospitals located in Turkey, namely, Mardin Training and Research Hospital (*n* = 21), Midyat Public Hospital (*n* = 6), and Kiziltepe Public Hospital (*n* = 3). Six healthcare staff refused to participate in this study. Mardin Training and Research Hospital is a public and tertiary hospital with a 700-bed capacity and around 150 ED healthcare staff. Midyat Public Hospital and Kiziltepe Public Hospital are public hospitals with 150-bed and 300-bed capacity, respectively. Convenience sampling was used to recruit participants. ED healthcare staff working in three hospitals were invited to participate in an interview. Those who accept to participate were included in the study. Participants characteristics were provided in Table 1.

**Table 1:** Participants characteristics

Participants	Occupation	Gender	Settings
Participant 1	Nurse	Male	Kiziltepe Public Hospital
Participant 2	Nurse	Female	Mardin Training and Research Hospital
Participant 3	Nurse	Female	Mardin Training and Research Hospital
Participant 4	Chief nurse	Male	Mardin Training and Research Hospital
Participant 5	Nurse	Female	Mardin Training and Research Hospital
Participant 6	Nurse	Male	Mardin Training and Research Hospital
Participant 7	ED physician	Female	Mardin Training and Research Hospital
Participant 8	ED consultant	Male	Mardin Training and Research Hospital
Participant 9	Nurse	Female	Mardin Training and Research Hospital
Participant 10	ED physician	Male	Mardin Training and Research Hospital
Participant 11	Nurse	Male	Mardin Training and Research Hospital
Participant 12	Nurse	Female	Mardin Training and Research Hospital
Participant 13	Nurse	Female	Mardin Training and Research Hospital
Participant 14	ED physician	Female	Kiziltepe Public Hospital
Participant 15	ED physician	Female	Kiziltepe Public Hospital
Participant 16	Nurse	Male	Mardin Training and Research Hospital
Participant 17	ED physician	Female	Mardin Training and Research Hospital
Participant 18	ED physician	Female	Mardin Training and Research Hospital
Participant 19	Nurse	Female	Mardin Training and Research Hospital
Participant 20	Nurse	Female	Mardin Training and Research Hospital
Participant 21	ED consultant	Female	Mardin Training and Research Hospital
Participant 22	Nurse	Male	Mardin Training and Research Hospital
Participant 23	Chief nurse	Female	Mardin Training and Research Hospital
Participant 24	Nurse	Male	Mardin Training and Research Hospital
Participant 25	Nurse	Female	Midyat Public Hospital
Participant 26	Nurse	Male	Midyat Public Hospital
Participant 27	Nurse	Female	Midyat Public Hospital
Participant 28	Nurse	Female	Midyat Public Hospital
Participant 29	ED physician	Male	Midyat Public Hospital
Participant 30	ED physician	Female	Midyat Public Hospital

### Procedure for the interviews

The interview guide (provided in Table 2) was developed by the researcher (A.B.) and piloted with 4 healthcare staff before commencing data collection. In-depth semi-structured interviews were conducted by researcher (A.B.), who had experience in conducting interviews and qualitative research, with 30 ED healthcare staff between 15/03/2022 and 30/04/2022. A quiet and comfortable private room at each hospital was arranged for the interviews. All interviews were audio-recorded and conducted in Turkish. The duration of the interviews ranged from 28 to 37 min. Data saturation was reached as no new information was gathered.

**Table 2:** Interview guide

Question 1	Emergency departments are the most important units where pandemics are managed. In this context, what changes have occurred in the emergency departments since the beginning of the COVID-19 pandemic? Can you explain?
Question 2	What kind of changes has the COVID-19 process caused in the functioning of emergency departments?
Question 3	What has the COVID-19 process changed in your work life? What were the challenges and differences arising from the pandemic?
Question 4	COVID-19 has a higher transmission rate than other diseases. We have lost many healthcare staff members in this pandemic. How did the fact that it was so contagious affect you psychologically and emotionally?
Question 5	How has the total number of visits to emergency departments changed during the COVID-19 pandemic? **Prompts**: Increased? Decreased? Same as usual?
Question 6	How has the number of “non-urgent” visits to emergency departments changed during the COVID-19 process? **Prompts**: Increased? Decreased? Same as usual?
Question 7	Can you tell me about the challenges faced by emergency department healthcare staff during the COVID-19 pandemic? **Prompts**: Risk of infection? Families of healthcare staff? Society’s perceptions of healthcare staff? Working conditions? etc.

### Ethical considerations

Ethical approval was obtained from the Mardin Artuklu University Non-Interventional Clinical Research Ethics Committee (Date: 08/03/2022, REF: E-76272411-900-47908). The participants were informed about the aim of the study, and verbal consent was obtained from all participants.

### Data analysis and rigor

The data were analyzed using thematic analyses. A six-step thematic analysis developed by Braun and Clarke [16] was followed: (1) familiarizing with the data, (2) generating initial codes, (3) searching for themes, (4) reviewing themes, (5) defining and naming themes, and (6) producing the report. NVivo software was used to manage the data analysis process. The researcher (A.B.) generates the themes and sub-themes by following a six-step thematic analysis. The developed sub-themes and themes were reviewed and checked by a second qualitative researcher (Y.Y.) using NVivo software, and consensus was reached by discussion. In addition, member check was carried out to ensure that the generated themes conformed to the participants’ views of the topic and the process, and therefore minimized researcher bias. Following the data analysis process, the researcher (A.B.) contacted five participants by email to compare how the themes generated from the analysis related to their experience (member check). They reported a high level of congruence (around 95%) between the themes descriptions and their views, thus adding credibility to the results. All of these processes add to the rigor of the results and increase their credibility and trustworthiness.

## Results

The thematic analysis was concluded with 15 sub-themes under three themes. The three themes were the impact of COVID-19 on emergency care services, the psychological effects of COVID-19 on ED healthcare staff, and the difficulties faced by ED healthcare staff.

### Impact of COVID-19 on emergency care services

This theme describes how COVID-19 changed the routine functioning of EDs, how ED visits were affected, how protective measures were taken, and how it affected the quality of care, resources used, and workload. Six sub-themes emerged: “introducing a COVID-19 unit in the ED”, “changes in the routine functioning of EDs”, “changes in the number of ED visits”, “quality of care”, “resources”, and “increased workload”.

#### Introducing a COVID-19 unit in the ED

A separate unit was introduced beside the ED to care for those with COVID-19 or suspected of COVID-19. Extra precautions against COVID-19 were taken in these units. Introducing these services might prevent contamination between those with and without COVID-19. In addition, the COVID-19 process delayed treatment in the ED. ED staff had to take precautions for all patients, which increased the time allocated for each patient and subsequently delayed treatment.

*“The COVID-19 unit was introduced in the ED. Those with suspected COVID-19 were referred to the COVID-19 unit for examination. COVID-19 tests were also conducted here.”* (Participant 16).

*“The ED has been divided into two units*,* the adult ED and the COVID-19 unit. The staff working in the COVID-19 unit paid attention to the use of masks*,* gowns*,* and visors*,* social distance*,* and cleaning rules while providing care to the patients.”* (Participant 19).

*“The COVID-19 unit was introduced. In the past*,* we used to quickly perform the procedures of the incoming patients without masks and gloves*,* but because of the pandemic*,* we started to perform the procedures of the patients by taking precautions. Because we did not know whether the incoming patients had COVID-19 or not*,* we took the same precautions for each patient.”* (Participant 5).

#### Changes in the routine functioning of EDs

The COVID-19 pandemic has changed the routine functioning of ED services. ED healthcare staff started to perceive all ED patients as suspected COVID-19 patients. Also, the ED healthcare staff had to take more precautions while examining the patients. They had to use protective equipment such as gloves, masks, aprons, and visors. In addition, healthcare staff also had to pay attention to social distancing and hygiene rules while providing care. All these extra precautions lead to fatigue and working under difficult working conditions, which could lead to high levels of stress and burnout. Working under such circumstances overwhelmed the ED healthcare staff during the COVID-19 pandemic.

*“We approached each patient as if they had COVID-19*,* without knowing whether the patients had COVID-19 or not. Also*,* it was very difficult to change the protective equipment each time.”* (Participant 16).

*“We started working with protective equipment. We were working more distantly with the patients. We have started to pay more attention to hygiene rules. The workload of the ED has increased enormously.”* (Participant 22).

The ED healthcare staff explained that they had difficulties dealing with COVID-19 as this was a new pandemic and it was an unknown process. ED healthcare staff start to use protective equipment and explain the importance of paying attention to prevention and precautionary methods to patients. In addition, ED healthcare staff had difficulties dealing with COVID-19 because of its high contagiousness.

*“Since it was the first time*,* we had experienced such a process*,* we had a lot of difficulties managing the process. The disease was new*,* and people were unconscious. Patients began to be treated more distantly and more carefully. We started to use protective equipment and made a lot of effort to explain the methods of protection and precaution to the patients. The contagiousness of the disease made our job even more difficult.”* (Participant 25).

*“We started using protective equipment to protect ourselves. Disinfectants were used. We pay attention to social distancing while examining patients. We changed the gloves frequently. I washed my hands often. Hand washing is essential. The risk of infection was very high. I started to use protective equipment regularly.”* (Participant 6).

#### Changes in the number of ED visits

ED healthcare staff stated that the number of ED visits by those whose condition was non-urgent decreased. The decrease in the number of ED visits could be a result of fear of acquiring COVID-19 infection and a desire to reduce the pressure on the ED. However, ED healthcare staff reported that patients started to visit the EDs again after a few months from the start of the pandemic. Such decrease in the number of non-urgent ED visits was temporary only at peak incidences of COVID-19.

*“At the beginning of the pandemic*,* non-urgent ED visits decreased. Patients did not come to the ED because of fear of COVID-19. However*,* they come to the ED now*,* it has become normal for the people*,* they do not care about COVID-19.”* (Participant 10).

*“In the early days of COVID-19*,* when there were restrictions*,* the number of patients with non-urgent conditions was quite low. When the restrictions were abolished*,* it started to increase again. The number of patients with non-urgent conditions increases when COVID-19 cases decrease*,* and the number of those with non-urgent conditions decreases when COVID-19 cases increase.”* (Participant 5).

#### Quality of care

ED healthcare staff stated that the COVID-19 pandemic decreased the quality of care in the ED because of the limited time allocated for each patient. This is an important issue for patient safety. ED healthcare staff stated that they had limited time for patient examination and this could affect the quality of care provided to patients.

*“There has been a decrease in the quality of treatment and care provided to patients. There has not been enough time for patients to be examined.”* (Participant 11).

#### Resources

ED healthcare staff stated that they experienced a lack of resources and sometimes unavailability of resources during COVID-19. ED healthcare staff stated that they sometimes had to work without protective equipment while dealing with patients with COVID-19. Working without protective equipment increases the risk of COVID-19 transmission. In addition, some medications were out of stock, and they had to use other available medications while caring for patients.

*“Protective equipment such as masks and gloves have decreased over time. Equipment began to be distributed in limited quantities per staff member. There were times when we had to work without protective equipment due to a lack of resources. This increased the risk of transmission of the disease.”* (Participant 12).

#### Increased workload

The participants stated that the workload of the ED increased during COVID-19. Increased workload decreases staff performance and efficiency. In addition, some of the staff were infected with COVID-19 and therefore unable to work during that time. This caused an increased workload for those who are not yet infected. Healthcare staff reported that they experienced irregular working hours due to an unpredicted number of staff to work, and this led to an irregular and limited social life. ED healthcare staff had to deal with COVID-19 with a limited number of staff. ED healthcare staff could not get enough rest, which caused staff burnout.

*“When we were infected with COVID-19*,* we were away from the hospital and from our work for a week. Therefore*,* there was a lack of staff and an increase in the workload. The staff had to work a 24-hour shift because employees with COVID-19 could not come to work. We had to work overtime. In the past*,* we worked according to a certain plan; however*,* there was an irregular working plan during the COVID-19 process. The high contagiousness of COVID-19 caused disruptions.”* (Participant 1).

*“Our working conditions have become very difficult. Our workload has increased considerably.”* (Participant 10).

*“The workload of the ED has increased with the pandemic. We could not get enough rest. There were limited number of staff. Our working life is always busy*,* stressful*,* and exhausting. I had a harder time with the disease. My work routine has changed. There was an unnecessary workload*,* but there was not enough healthcare staff.”* (Participant 26).

### Psychological effects of COVID-19 on ED healthcare staff

This theme explains how the COVID-19 pandemic affects ED healthcare staff, including their social life, fears, psychological and emotional effects, and how they were supported during COVID-19. Six sub-themes emerged: “Staying away from family”, “fear”, “society’s perspective on healthcare professionals”, “morale-staff burnout”, “psychological and emotional effects” and “unable to receive enough support”.

#### Staying away from family

Almost all participants reported that they had experienced difficult times because they had to stay away from their families to reduce the risk of infection and keep them safe. Staying away from their families affects ED healthcare staff psychologically and increases their anxiety and stress levels.

*“During the COVID-19 pandemic*,* most of the staff did not go home; they stayed away from their families because of the risk of infection. Some people stayed in hotels and other types of accommodation”* (Participant 10).

*“The time we spend with our families has decreased. The high contagiousness of the disease and our fear of infecting our loved ones increased our anxiety and stress levels. We had to be very careful not to get the disease and infect our loved ones.”* (Participant 11).

*“Being away from my family made me psychologically depressed.”* (Participant 16).

#### Fear

The participants stated that they experienced fear regarding COVID-19. Such fear was regarding their own health and the potential consequences of contracting COVID-19, hospitalization and transmitting the virus to others, including colleagues, patients, and family members. Participants stated that the pandemic affected their mental well-being. Many healthcare staff faced considerable stress and anxiety during COVID-19.

*“At the beginning of the pandemic*,* we were all psychologically feared. I was worried and afraid of how the disease would progress*,* how I would pass it if I infected*,* whether I would be hospitalized*,* and whether it would infect others if it infected me.”* (Participant 16).

*“Psychologically*,* we were in a constant state of fear.”* (Participant 24).

*“It affected me badly. I am a person who loves to live. I was afraid of dying. The pandemic process was very difficult and worn me out.”* (Participant 26).

*“When there are deaths or negative situations among our colleagues*,* we are inevitably affected emotionally. We were affected emotionally. Also*,* I was afraid of infecting my family or any other person”* (Participant 1).

#### Society’s perspective on healthcare professionals

Almost all healthcare staff stated that they were excluded from the members of the society because of being a healthcare staff. Being healthcare staff during the pandemic times means that they have a higher risk of transmission of the disease, and therefore, they were not welcomed in society. Healthcare staff felt excluded by members of society during the pandemic. Such exclusion by society affects the mental health and morale of healthcare staff.

*“Society ran away from us during the pandemic.”* (Participant 10).

*“In this process*,* everyone treated us as if we had COVID-19 because we were healthcare professionals. I felt so excluded.”* (Participant 11).

*“Because I was a healthcare staff member*,* even my neighbours were not close to me*,* and they did not even want to use the same lift.”* (Participant 13).

*“Society has become afraid of us.”* (Participant 14).

#### Morale-staff burnout

Some ED healthcare staff members were psychologically affected. ED healthcare staff struggled with COVID-19 and experienced sleeplessness, stress, and exhaustion. Participants reported a high level of burnout related to COVID-19. It was found that COVID-19 caused an increased workload in the ED, which led to staff being exhausted, getting stressed, working without getting enough sleep, and experiencing burnout.

*“In addition to the serious battle we fought physically*,* we were also fighting a great battle spiritually. We had many friends whose psychology was disturbed by this disease. As a result*,* we were constantly tensed and stressed. We experienced burnout in the process.”* (Participant 25).

*“Due to the increasing number of cases*,* our workload in the ED has increased a lot. This made it very difficult for us; we were sleepless for days.”* (Participant 27).

*“I cried for nights because of the difficulty of this process. It was an exhausting process. Understanding and tolerance are always expected from us*,* but we are never shown these things. We are human too*,* so we get burnout*,* angry*,* and tired.”* (Participant 30).

*“Healthcare staff who contracted COVID-19 had to start work after a few days without being tested again.”* (Participant 24).

*“This process has demoralized all of us. Our staff and friends have been infected with COVID-19. Some of the healthcare staff members died. We have also experienced such incidents. We are very saddened by these events.”* (Participant 4).

#### Psychological and emotional effects

Participants expressed that the pandemic had a significant negative impact across multiple domains of life, including family, social, and work life. Participants reported that their social life was negatively affected. They experienced feelings of isolation or disconnection from friends, extended family, or social networks. Such social isolation and working under difficult circumstances overstressed ED healthcare staff and, affected them psychologically and emotionally. ED healthcare staff stated that such conditions increased their stress levels.

*“Our family life*,* social life*,* and working life have been affected in a very negative way.”* (Participant 25).

*“It was a very difficult process*,* and we were affected psychologically.”* (Participant 28).

*“It wore me out psychologically. My social life was affected too much. I could not see my loved ones outside.”* (Participant 8).

*“We had difficult days psychologically and physically”* (Participant 12).

*“The pandemic has affected us negatively. We lost a chief physician who was a former colleague of mine. I was affected by this loss. When we thought about the risk of our family*,* relatives*,* and those with chronic diseases getting COVID-19*,* we were burned out more.”* (Participant 13).

#### Unable to receive sufficient support

Some ED healthcare staff reported that they did not receive sufficient support during the COVID-19 pandemic. Their motivation decreased due to unable to receive the required support. They experienced violence and were affected financially and spiritually. In addition, they experienced social isolation and a lack of social life. Accumulation of all these negative conditions affects ED healthcare staff psychologically and leads them to feel alone. The ED healthcare staff described that the absence of support and motivation contributed to their ability to cope with the challenges they faced.

*“This process was difficult. There was no source of motivation. During this period*,* I was separated from my family and friends. I had difficulties both financially and spiritually. Cases of verbal and physical violence have increased. Health policies must be changed.”* (Participant 30).

### Difficulties faced by the ED healthcare staff

This theme describes the difficulties faced by ED healthcare staff during the COVID-19 pandemic. Three sub-themes emerged “difficult working conditions”, “community-based effect difficulties”, and “COVID-19 was an unknown situation”.

#### Difficult working conditions

ED healthcare staff worked under difficult working conditions during COVID-19. They had to work by using protective equipment all the time while working. In addition, ED staff stated that the risk of infection for them was high, and this caused them to be stressed, stay away from their families, and be isolated from society.

*“We had a lot of trouble. We had to work with heavy protective equipment. The risk of infection was very high. As the number of cases increased*,* patient circulation increased*,* which affected us negatively.”* (Participant 1).

*“We tried to be more careful while working. Healthcare staff are faced with difficulties due to a lack of protective equipment. We put in more effort. We sweated while working with protective equipment. There have also been times when we have put our health at risk.”* (Participant 12).

*“It was very difficult to work with protective equipment; standing with them all day long left us drenched in sweat. It was a very difficult process*,* and it affected us psychologically.”* (Participant 28).

#### Community-based effect difficulties

The ED healthcare staff stated that they faced significant difficulties due to non-compliance with safety standards by members of the community. ED healthcare staff reported members of the community did not adhere to recommended safety measures such as wearing masks, maintaining social distance, and practising proper hygiene. This non-compliance could cause serious risks for both healthcare staff and other patients because it increases the likelihood of virus transmission within healthcare settings.

*“We had serious problems because the patients did not comply with the rules of masking*,* social distance*,* and hygiene.”* (Participant 10).

*“When patients are referred to the COVID-19 unit*,* they do not go there to avoid testing. We started missing real emergency cases. Patients with high blood pressure*,* heart failure*,* and diabetes started to burden the emergency department because they did not use their medications or because their controls were delayed.”* (Participant 10).

#### COVID-19 is an unknown situation

The participants stated that the COVID-19 pandemic was an unknown situation and, therefore, they did not know how to respond to this pandemic, including the symptoms of the disease, how to approach patients, what precautions should be taken, and how to treat or alleviate the disease.

*“At the beginning of the pandemic*,* we did not know exactly what to do because the disease was new. Information was limited. Therefore*,* the emergency department could not be managed.”* (Participant 2).

*“Since we were caught unprepared at the beginning of the pandemic*,* we had difficulties about what to do*,* how to take actions*,* how to make the patient use the protective equipment*,* and how to protect ourselves.”* (Participant 18).

*“Because we had no information about the disease*,* we had difficulties in controlling the process*,* and we did not know how to approach the patients.”* (Participant 22).

*“Since we have experienced such a process for the first time*,* we have had many difficulties in managing the process. The disease was new*,* and people were unconscious. All patients were approached with suspicion of COVID-19. Patients began to be treated more distantly and more carefully.”* (Participant 25).

*“We did not know what the disease was like at the beginning of the pandemic and how we should approach patients. Therefore*,* the care and treatment provided to patients was inadequate.”* (Participant 28).

## Discussion

This study found that COVID-19 negatively affects emergency care services in Turkey, including changes in the functioning of EDs and ED visits, decreased quality of care, increased use of resources, and workload. In line with the existing literature [2, 17–20], this study revealed that the number of ED visits decreased at the beginning of the pandemic. The existing literature showed that the number of ED visits decreased by 65% [20], by 50% [21], and by 37% [22] during the first lockdown. Such a reduction in the number of ED visits could be a result of restrictions and the fear of being infected with COVID-19. The existing literature showed that patients were concerned about visiting the ED during the COVID-19 pandemic [23]. However, ED visits enormously increased after abolishing the restrictions as patients adapted their health-seeking behaviors throughout the pandemic.

The results of this study highlighted that ED healthcare staff experienced many difficulties during the COVID-19 pandemic, such as staying away from their family, fear, negative society perspective on healthcare staff, morale and staff burnout, psychological and emotional effects, and inability to receive enough support. This study found that ED healthcare staff had to stay away from their families during the COVID-19 pandemic because of the risk of infection to their families, which concurred with the existing literature [24, 25]. This affects ED healthcare staff psychologically and increases their anxiety and stress levels. In addition, this study found that ED healthcare staff experienced distress and a high level of burnout during the COVID-19 pandemic, which concurs with some of the existing studies [8, 26–30]. In line with some studies [26, 31, 32], this study found that experiencing burnout could be negatively associated with patient satisfaction, quality of care, staff morale and retention, and therefore a loss of workforce for the future.

In line with the results of this study, ED healthcare staff faced challenges such as increased workload and resource constraints during the COVID-19 pandemic [10, 33–35]. ED healthcare staff face mental burdens and physical exertion when caring for patients in the ED during the COVID-19 pandemic. ED healthcare staff provide care under difficult circumstances with limited resources. In line with these results, some studies suggested that stress factors in relation to providing health care for patients with COVID-19 should be addressed [7, 36].

### Strengths and limitations

One of the strengths of this study is to include a large sample of ED healthcare staff working in three different hospitals with in-depth semi-structured interviewing resulting in a rich and detailed source of qualitative data for analysis. One of the limitations of this study could be the generalizability of the results due to the nature of qualitative research, which does not attempt to generalize the results to other populations. In addition, this study was conducted around 1 year later than the starting point of time of COVID-19. Therefore, participants may not recall all their experiences during COVID-19. The results may be transferrable to policymakers, ED directors, or other key stakeholders across Turkey and other countries with similar contexts.

## Conclusion and recommendations

This study could provide a better understanding of how ED services and ED staff were affected by the COVID-19 pandemic, including decreased quality of care in the ED, increased workload, resource strains, psychological effects on ED staff, and related difficulties. Learning from such lived experiences and developing appropriate interventions to minimize the difficulties faced during COVID-19 would allow better management of future pandemics.

Staff burnout threatens the quality of patient care and staff retention, and therefore this should be addressed by ED directors and leaders. Supporting staff in dealing with difficulties such as psychological problems, fears, burnout and providing a safe working environment could contribute to staff well-being, a better workforce for the future, and staff retention. This study calls for a reform to address the challenges faced by healthcare staff, improve the overall response to public health crises such as the COVID-19 pandemic, and enhance the resilience of healthcare systems despite future crises. This study could inform appropriate stakeholders regarding lessons learned from COVID-19 to better manage future pandemics. Learning from such lived experiences and developing appropriate interventions to minimize the difficulties faced during COVID-19 would allow better management of future pandemics.

## Data Availability

No datasets were generated or analysed during the current study.
